# Supplementary material for: Structural basis for metal ion transport by the human SLC11 proteins DMT1 and NRAMP1
Source: Nat Commun. 2025 Jan 17;16:761. doi: 10.1038/s41467-024-54705-0 (PMC11742427; doi:10.1038/s41467-024-54705-0)
Supplement: Supplementary file 1 — Supplementary Information [file 41467_2024_54705_MOESM1_ESM.pdf]

Supplementary Information

Structural basis for metal ion transport by the human SLC11 proteins DMT1 and NRAMP1

Márton Liziczai, Ariane Fuchs, Cristina Manatschal and Raimund Dutzler

Supplementary Figures

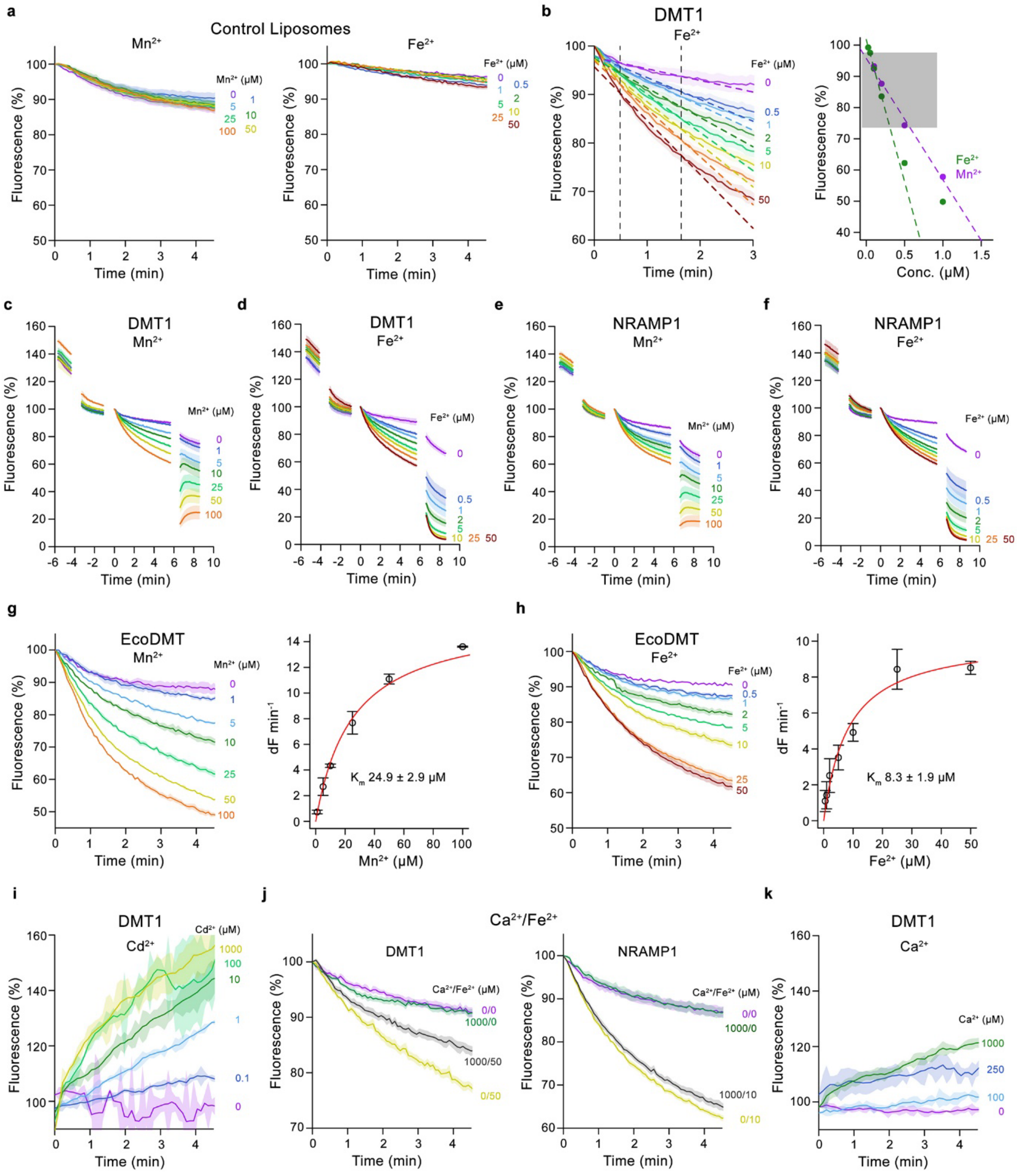

Supplementary Fig. 1: Metal ion transport. a Time and concentration dependent fluorescence decrease of the fluorophore calcein trapped inside vesicles not containing a reconstituted transport protein upon addition of Mn^{2+} (left) or Fe^{2+} (right) to the outside buffer shown as negative control. Data display mean of seven (Mn^{2+}) and four (Fe^{2+}) experiments from two and one independent liposome preparations, respectively. **b** Time and concentration dependence of Fe^{2+} uptake into proetoliposomes containing reconstituted DMT1 assayed by the quenching of the fluorophore calcein trapped inside the liposome. Left panel shows the indicated region of Fig. 1c with the transport rate approximated by the difference in fluorescence 30 and 90 seconds after start of the transport measurement (16

measurement points) following the addition of the metal ions indicated by dashed lines. Right panel shows the linear dependence between fluorescence quenching and metal ion concentration in the investigated range. **c-f** Full traces of transport experiments shown in Fig. 1a-d. **c** DMT1 mediated Mn^{2+} transport, **d** NRAMP1 mediated Mn^{2+} transport, **e** DMT1 mediated Fe^{2+} transport, **f** NRAMP1 mediated Fe^{2+} transport. After two minutes of fluorescence recording an inwardly directed negative membrane potential was established by addition of the ionophore valinomycin (t=-4 min). Metal ions were added after equilibration of the signal (t=-30 s) and the transport mediated decay was recorded for six minutes (from t=0 to 6 min), before the addition of the ionophore calcimycin to equilibrate the metal ion concentration. Data show seven experiments from two independent reconstitutions for Fe^{2+} transport by DMT1, eight experiments from two independent reconstitutions for all other experiments, errors are s.e.m. **g, h** Transition metal ion transport into proteoliposomes by the prokaryotic homologue EcoDMT. Time and concentration dependence of metal ion uptake is shown on the left. Right panels display fit of initial transport rates to a Michaelis-Menten equation with respective K_m values indicated: **g** Mn^{2+} and **h** Fe^{2+} transport. Data show mean of two (Mn^{2+}) and three (Fe^{2+}) experiments form one reconstitution. **i** Cd^{2+} transport into proteoliposomes containing DMT1. **j** Interference of Fe^{2+} transport into proteoliposomes containing DMT1 (left), or NRAMP1 (right) by Ca^{2+}. **k** Addition of Ca^{2+} to proteoliposomes containing DMT1 did not show a pronounced change, suggesting that there is no transport of the divalent metal ion. Data show mean of eight experiments from two reconstitutions. **c-h, j** Uptake of Mn^{2+} and Fe^{2+} was assayed by the quenching of the fluorophore calcein trapped inside the vesicles. **i**, **k** Experiments assay the Cd^{2+} and Ca^{2+} concentration dependent fluorescence increase of the fluorophore Fura-2 trapped inside the vesicles. **i-j** Data show mean of three and four experiments from one reconstitution, respectively. **a-k** errors are s.e.m. for n>2. Fluorescence is normalized to the value after addition of substrate (t=0). Metal ion concentrations are indicated.

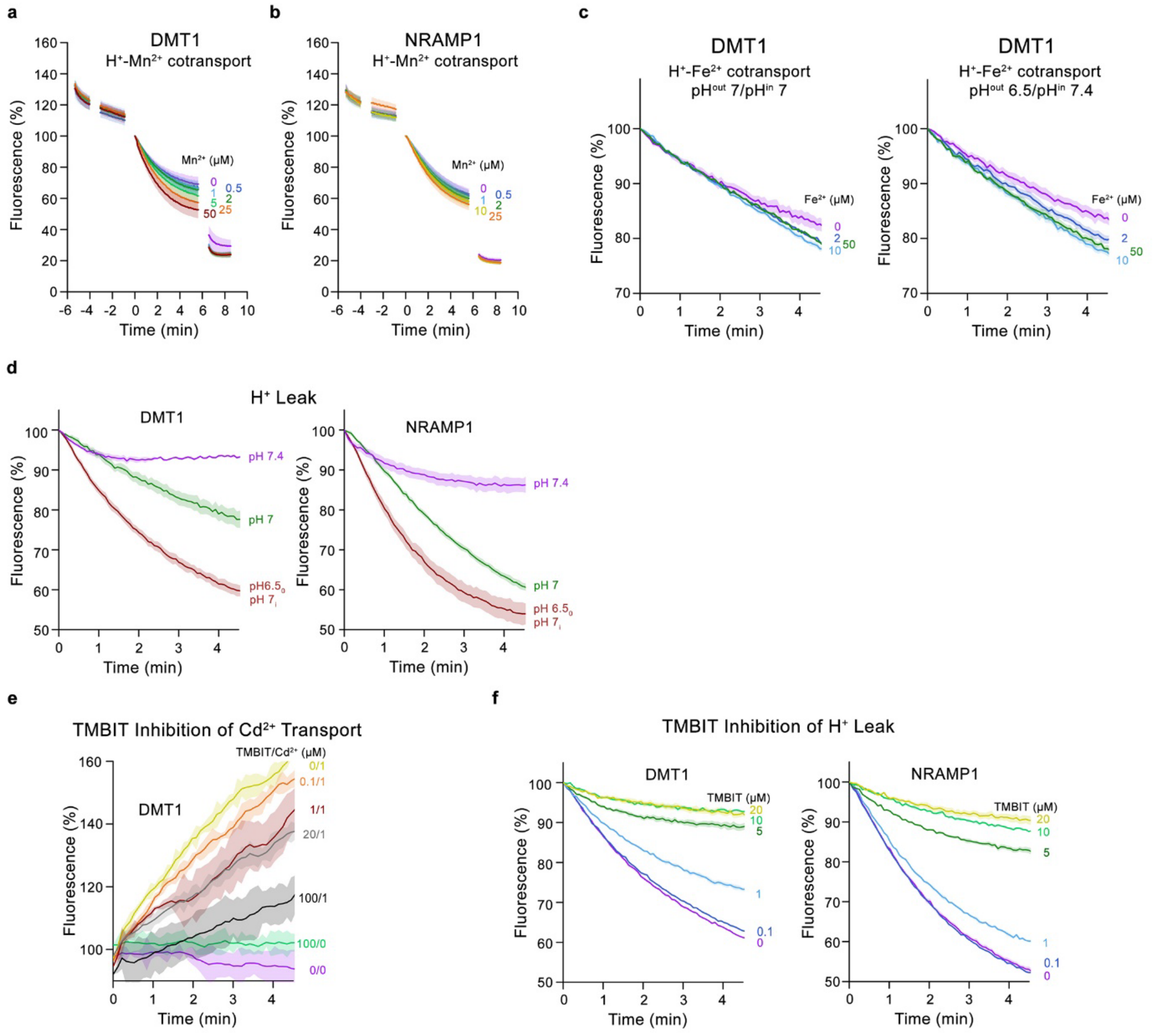

Supplementary Fig. 2: Proton transport. a, **b** Full traces of transport experiments shown in Fig. 2a, b. **a** Mn^{2+} driven H^+ transport mediated by DMT1, **b** Mn^{2+} driven H^+ transport mediated by NRAMP1. After two minutes of fluorescence recording metal ions were added (at t=-4 min) before establishment of an inwardly directed negative membrane potential by addition of the ionophore valinomycin (t=-30 s) and the transport mediated decay was recorded for six minutes (from t=0 to 6 min), before the addition of the ionophore CCCP to equilibrate the proton concentration. Data show mean of seven and six experiments from two reconstitutions for DMT1 and NRAMP1 respectively. **c** Fe^{2+} driven H^+ transport mediated by DMT1, assayed at symmetric pH 7.0 (left) or at an outside pH of 6,5 (right). The initial pH inside proteoliposomes was set to 7.4. Data show mean of four experiments from one reconstitution, errors are s.e.m. **d** pH-dependence of the uncoupled H^+ leak in DMT1 (left) and NRAMP1 (right). Traces show H^+ uptake at symmetric pH 7.4 (magenta) and pH 7.0 (green) and under asymmetric conditions with pH 6.5 on the outside and 7.0 on the inside of liposomes (brown). **e** Concentration dependent inhibition of Cd^{2+} transport into proteoliposomes containing DMT1 by TMBIT added to the outside. **f** Concentration dependent inhibition of the metal ion independent H^+ leak upon addition of TMBIT to the outside of proteoliposomes containing DMT1 (left) and NRAMP1 (right). H^+ flux was assayed under asymmetric conditions with pH 6.5 on the outside and 7.0 on the inside of liposomes. **a-d**, **f** H^+ transport

was assayed by the quenching of the fluorophore ACMA. **e** Experiments assay the Cd^{2+} dependent fluorescence increase of the fluorophore Fura-2 trapped inside the vesicles. **d-f** Data show mean of four experiments from one reconstitution. **a-f**, errors are s.e.m. Fluorescence is normalized to the value after addition of substrate (t=0). Substrate and inhibitor concentrations are indicated.

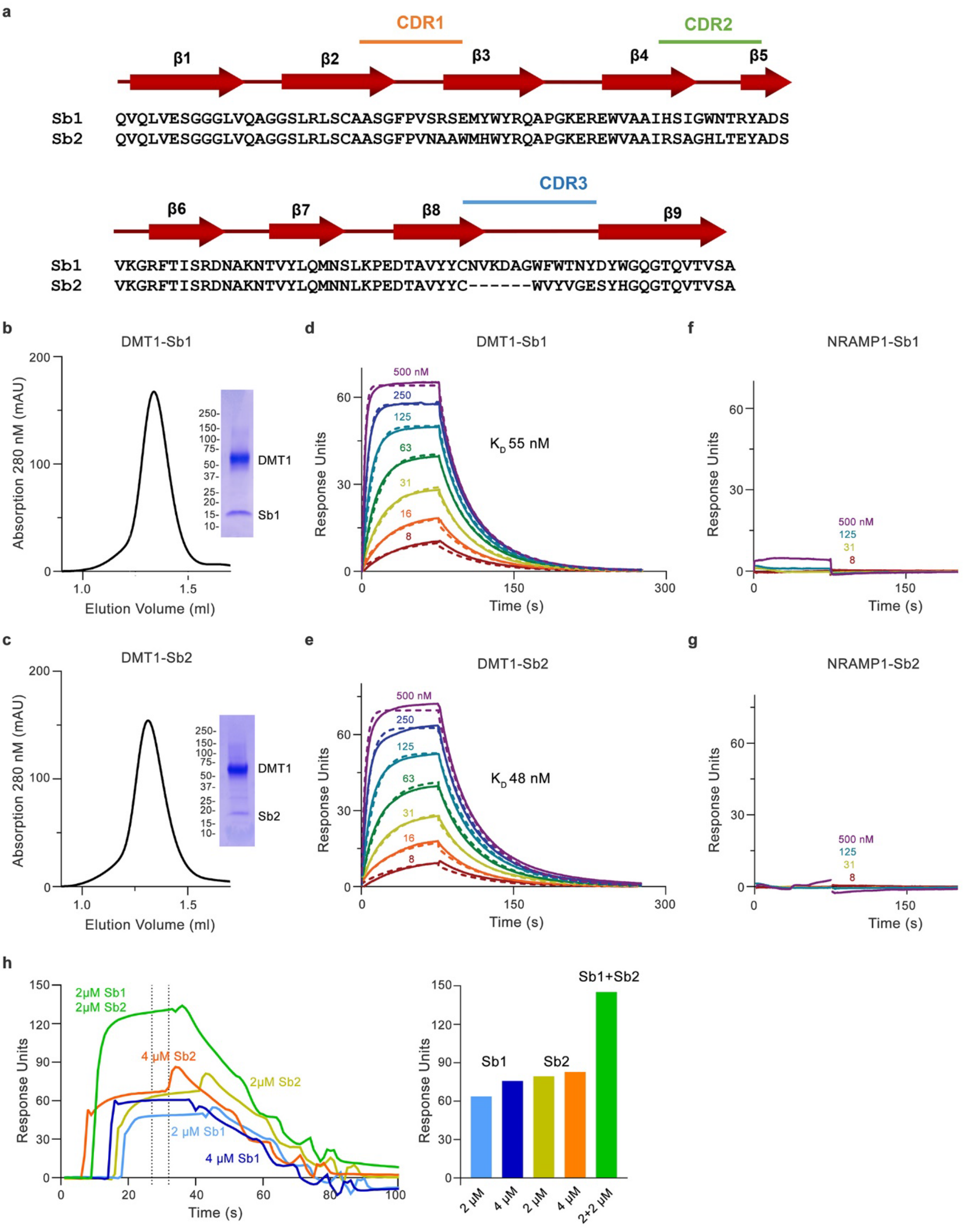

Supplementary Fig. 3: Sybody selection and characterization. **a** Sequence alignment of the two DMT1 binders Sb1 and Sb2. Secondary structure is shown above. The locations of complementary determining regions (CDRs) are indicated. **b**, **c** SEC profiles of the purified DMT1/Sb1 (**a**) and DMT1/Sb2 complex (**b**). Insets show SDS-PAGE gels with molecular weights (kDa) indicated. The bands above 50 kDa correspond to DMT1, the bands between 15-20 kDa to the respective sybody. **d**, **e** Affinity determination by SPR experiments using immobilized DMT1 and varying concentrations of Sb1 (**d**), Sb2 (**e**). Individual traces of the association and dissociation of sybodies and dashed lines representing the fit to a 1:1 binding model are shown in distinct colors. K_D values are indicated. **f**, **g** SPR experiments using immobilized NRAMP1 and varying concentrations of Sb1 (**f**), Sb2 (**g**) do not show any indication for a specific interaction. **h** Left, response upon application of individual sybodies at indicated concentrations and joint application of both sybodies indicate simultaneous binding of Sb1 and Sb2 sybodies to DMT1. Dashed lines indicate region that was averaged and displayed in the bar diagram, right.

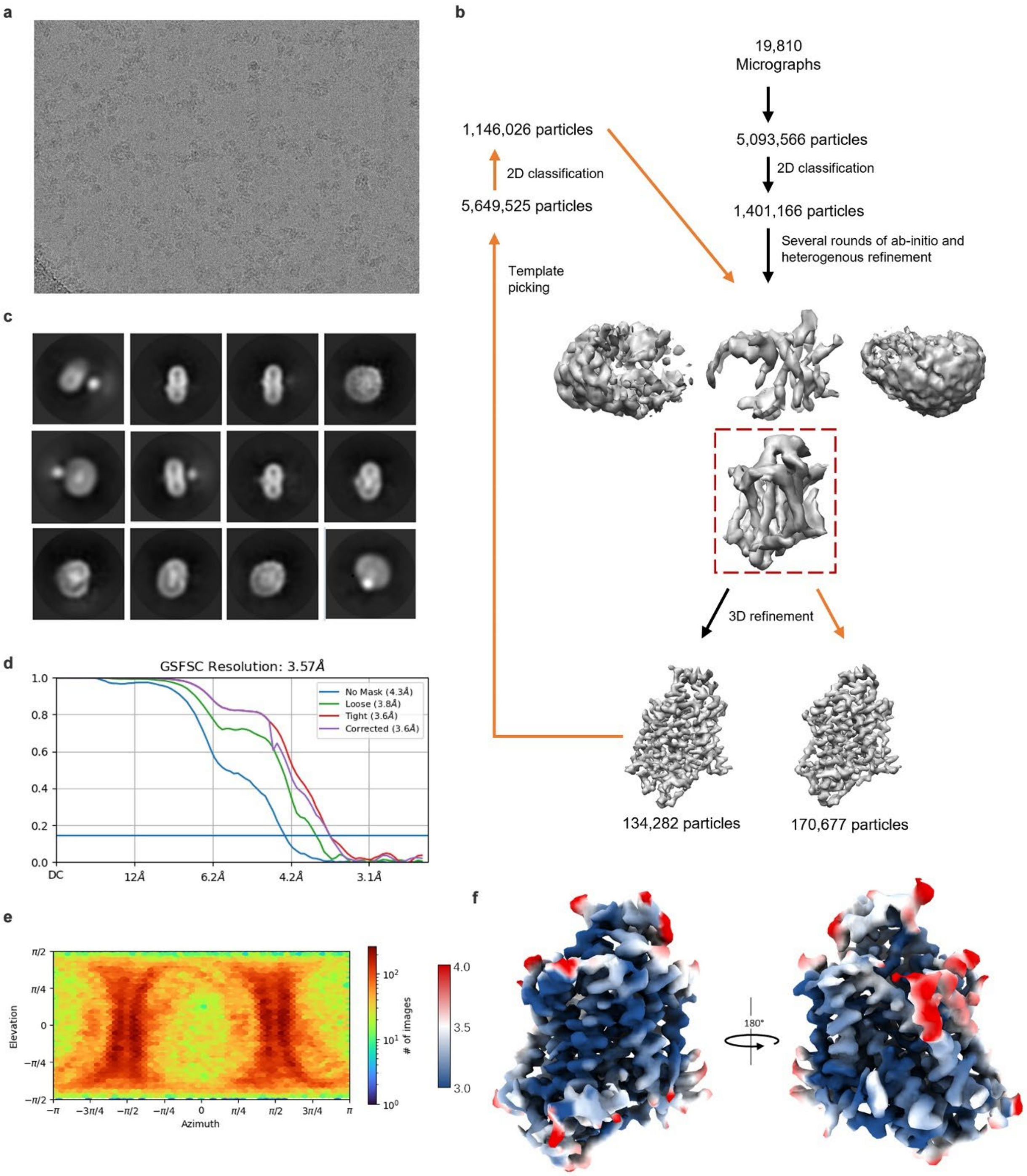

Supplementary Fig. 4: CryoEM reconstruction of the DMT1/Sb2 complex. a Representative motion corrected micrograph for the dataset of the DMT1-Sb2 complex. **b** Data processing workflow. Particles were extracted, subjected to several rounds of 2D classification and one ab-initio reconstruction with several classes. Particles were sorted between a high-quality class containing DMT1 particles (boxed) and 3 classes of particles not containing the protein via several rounds of heterogenous refinement until the resolution of the DMT1 class did not improve any further. The final set of particles were refined by non-uniform refinement, 2D templates were generated which were subsequently used for template picking. The new particles were extracted, subjected to several rounds of 2D classification and heterogenous refinement using the previously generated volumes. The final set of particles were refined

by non-uniform refinement and subsequent local refinement. **c** Representative 2D class averages. **D** GS-FSC plot of the final reconstruction reaching a nominal resolution of 3.6 Å. **e** Direction distribution of particles contributing to the final refinement. **f** Final map colored according to local resolution.

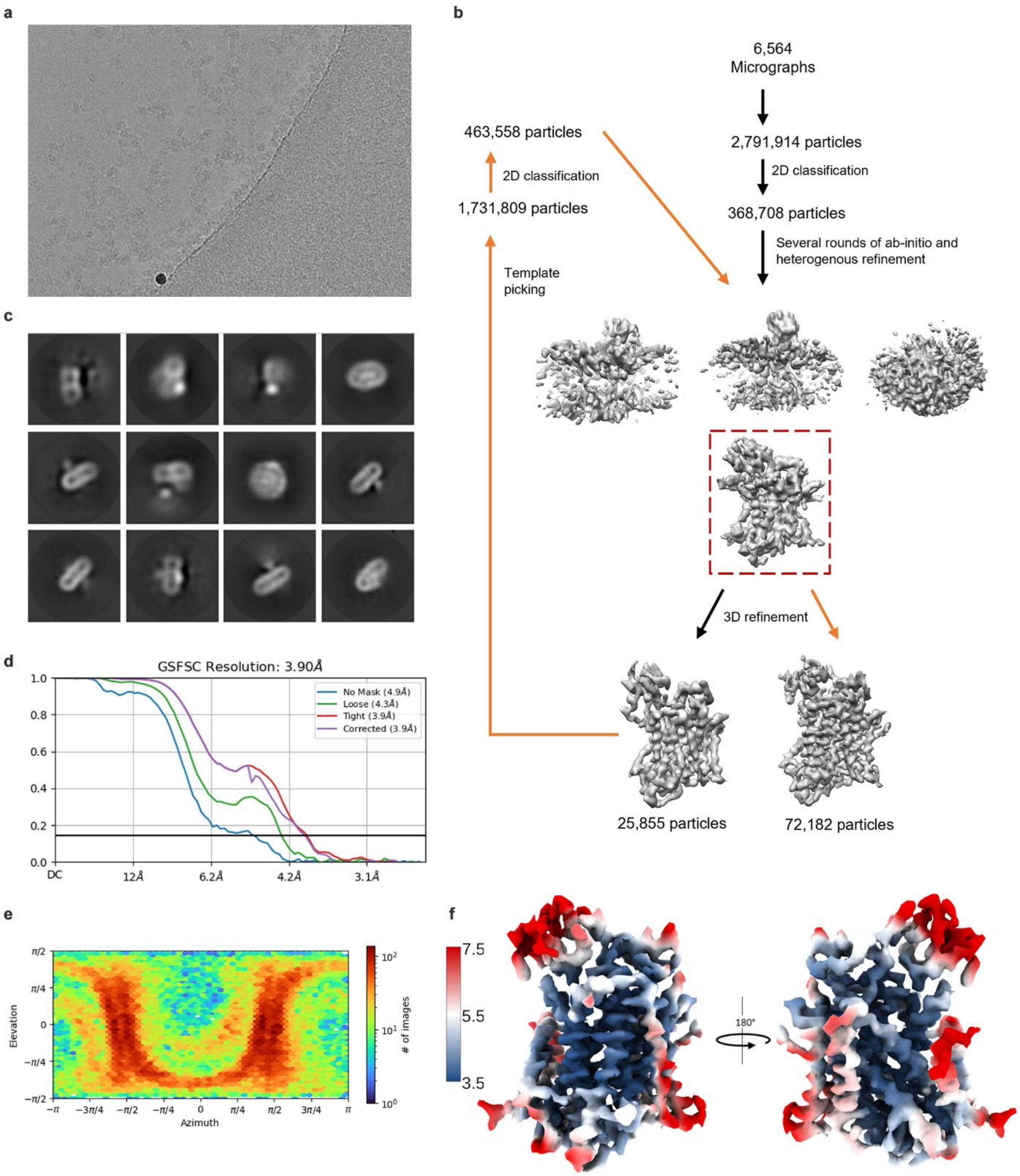

Supplementary Fig. 5: CryoEM reconstruction of the DMT1/Sb1/Sb2 complex. a Representative motion corrected micrograph of the dataset of the DMT1-Sb1-Sb2 complex. **b** Data processing workflow. Particles were extracted, subjected to several rounds of 2D classification and multiple ab-initio reconstructions with several classes. Particles were sorted between a class containing high quality particles (boxed) and 3 low quality classes via several rounds of heterogenous refinement until the

resolution of the class resembling DMT1 did not improve any further. The final set of particles were refined by non-uniform refinement, 2D templates were generated which were used for template picking. The new particles were extracted, subjected to several rounds of 2D classification and heterogenous refinement using the previously generated volumes. The final set of particles were refined by non-uniform refinement and subsequent local refinement. **c** Representative 2D class averages. **d** GS-FSC plot of the final reconstruction reaching a nominal resolution of 3.9 Å. **e** Direction distribution of particles included in the final refinement. **f** Final map colored according to local resolution.

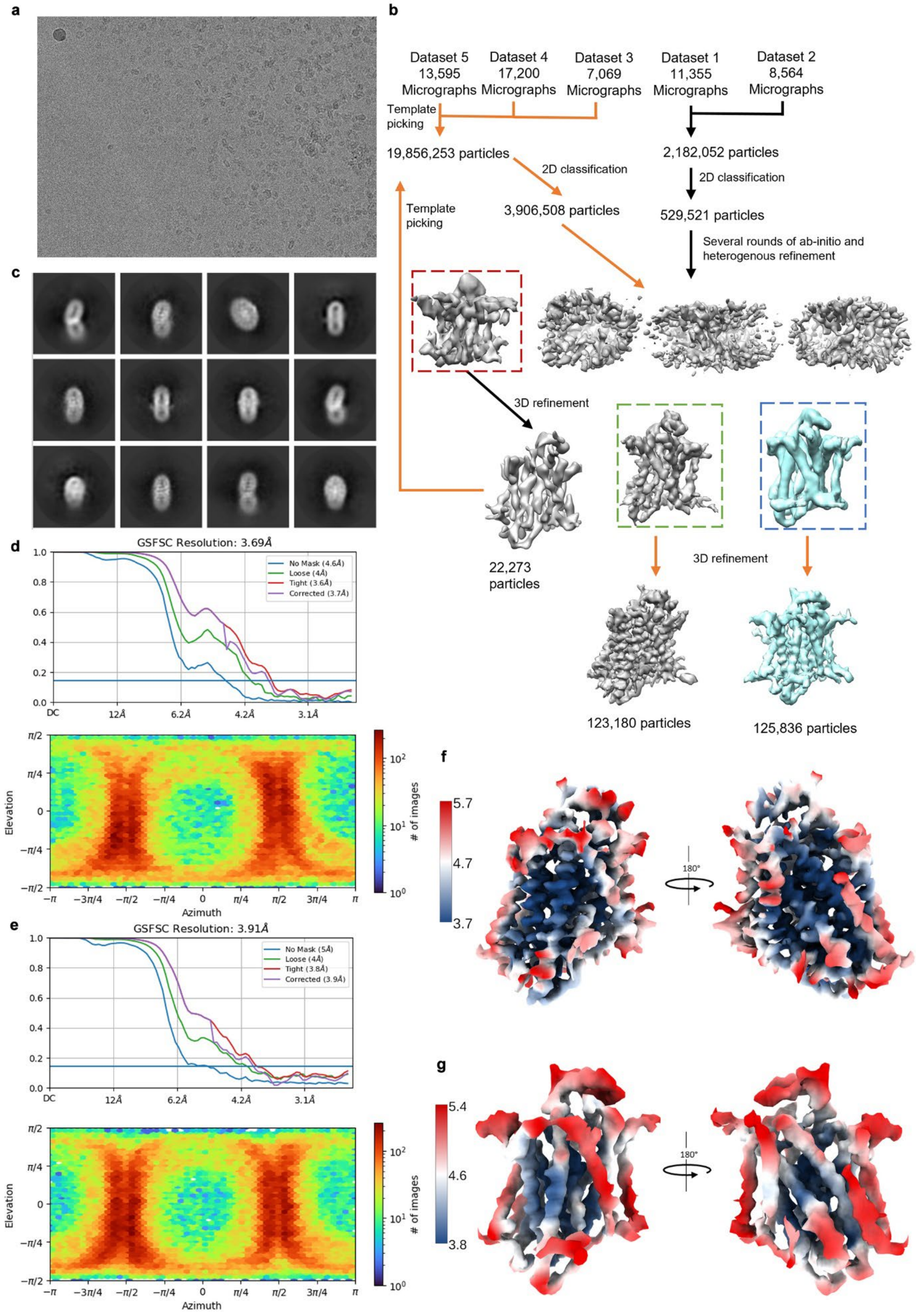

Supplementary Fig. 6: CryoEM reconstruction of NRAMP1. a Representative motion corrected micrograph of the NRAMP1 dataset. **b** Data processing workflow. Particles were extracted from 2

datasets, subjected to several rounds of 2D classification and multiple ab-initio reconstructions with several classes. Particles were sorted between a class containing high quality particles (red box) and 3 classes not containing protein particles via several rounds of heterogenous refinement until the resolution of the high-quality class did not improve any further. The final set of particles was refined by non-uniform refinement and the resulting volume was used to generate 2D templates, which were subsequently used for template picking from all 5 datasets. The new particles were extracted, subjected to several rounds of 2D classification and heterogenous refinement using the previously generated volumes. Through new ab-initio reconstructions, two new promising 3D volumes emerged, one resembling an inward-open confirmation (green box, $NRAMP1^{inw}$) and one in an occluded state (blue box, $NRAMP1^{occ}$). Particles were sorted between the two new high-quality classes (green and blue box) and classes of low-quality particles by heterogenous refinement. The final set of particles from both volumes were refined by non-uniform refinement and subsequent local refinement. **c** Representative 2D class averages. **d** GS-FSC plot of the final reconstruction reaching a resolution of 3.7 Å (top) and direction distribution of particles in the final refinement (bottom) of $NRAMP1^{occ}$. **e** GS-FSC plot of the final reconstruction reaching a resolution of 3.9 Å (top) and the direction distribution of particles in the final refinement (bottom) of $NRAMP1^{inw}$. Final map colored according to local resolution of NRAMP1 in the **f,** occluded and **g,** inward open conformation.

a

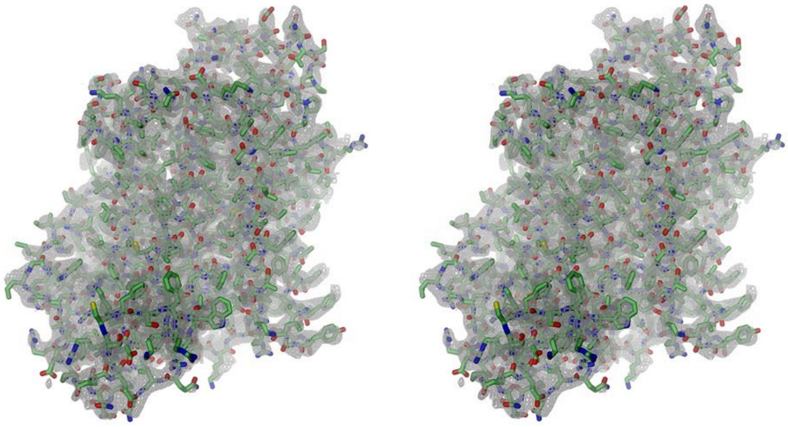

b

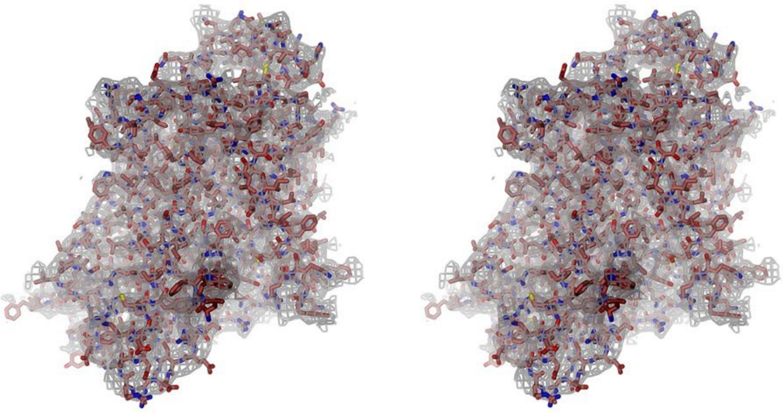

Supplementary Fig. 7: Stereo views of the cryo-EM densities of different SLC11 structures. a Cryo-EM density of the DMT1/Sb2 complex at 3.6 Å (contoured at 7σ) superimposed on the model shown as sticks. The density of the sybody Sb2 was not resolved in the map. **b** Cryo-EM density of NRAMP1 at a nominal resolution of 3.7 Å (contoured at 7σ) superimposed on the model shown as sticks.

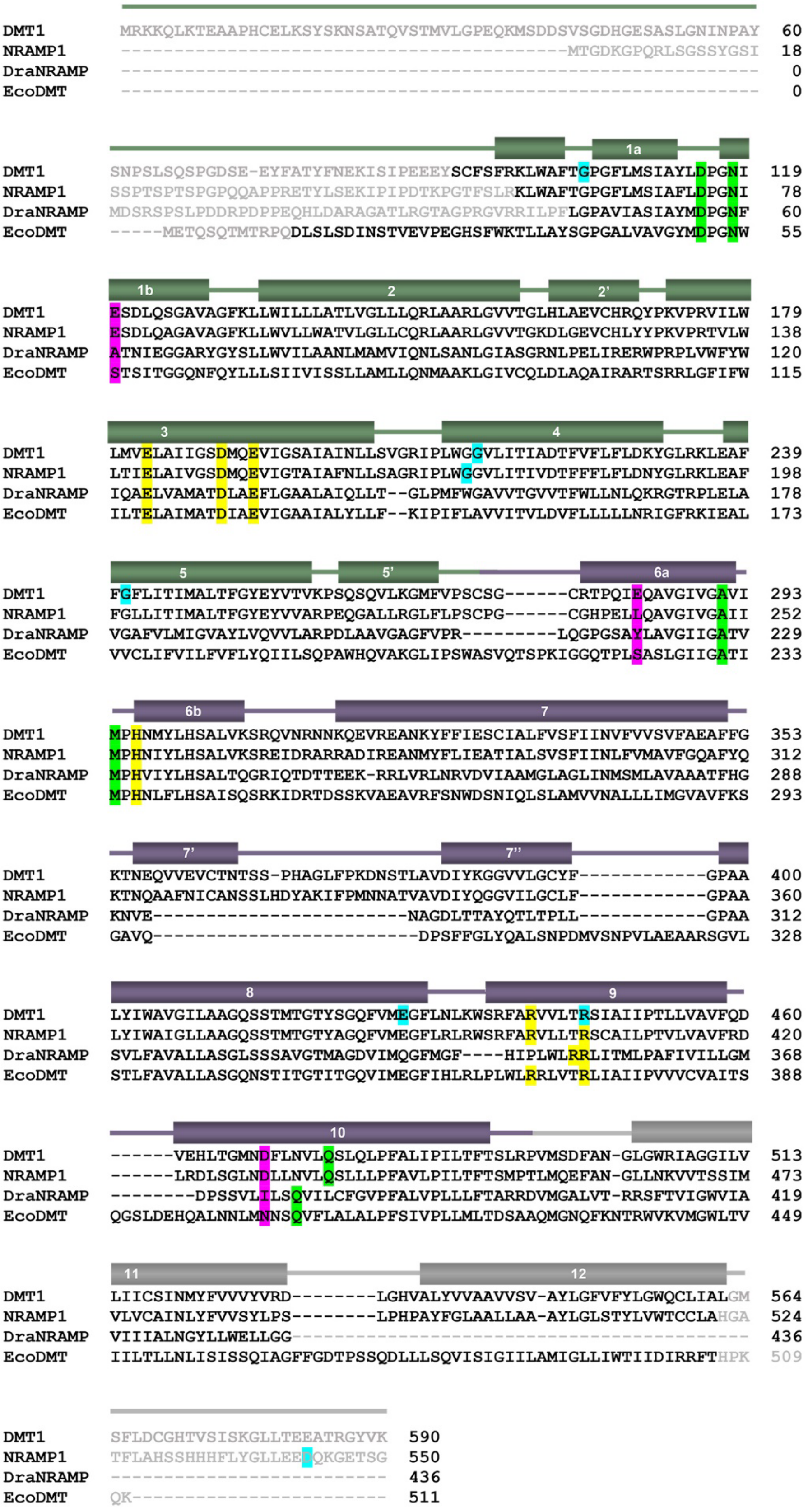

Supplementary Fig. 8: Sequence alignment of SLC11 homologues. Sequence alignment of the human transporters DMT1 (UniProtKB identifier P49281-3) and NRAMP1 (UniProtKB identifier P49279-

1) and their prokaryotic homologs EcoDMT (UniProtKB identifier E4KPW4) and DraNRAMP (UniProtKB identifier Q9RTP8). Secondary structure elements of DMT1 are shown above the sequences. The N-terminal repeat (α-helices 1–5) is colored in green, the C-terminal repeat (α-helices 6–10) in blue, α-helices 11 and 12 in grey. Selected residues are highlighted: green, ion-binding site, yellow, residues proposed to contribute to H^+ coupling, magenta, residues at the extracellular side of DMT1 that might form part of an inhibitory Ca^{2+} binding site, cyan, location of disease-causing mutations of residues of DMT1 and NRAMP1.

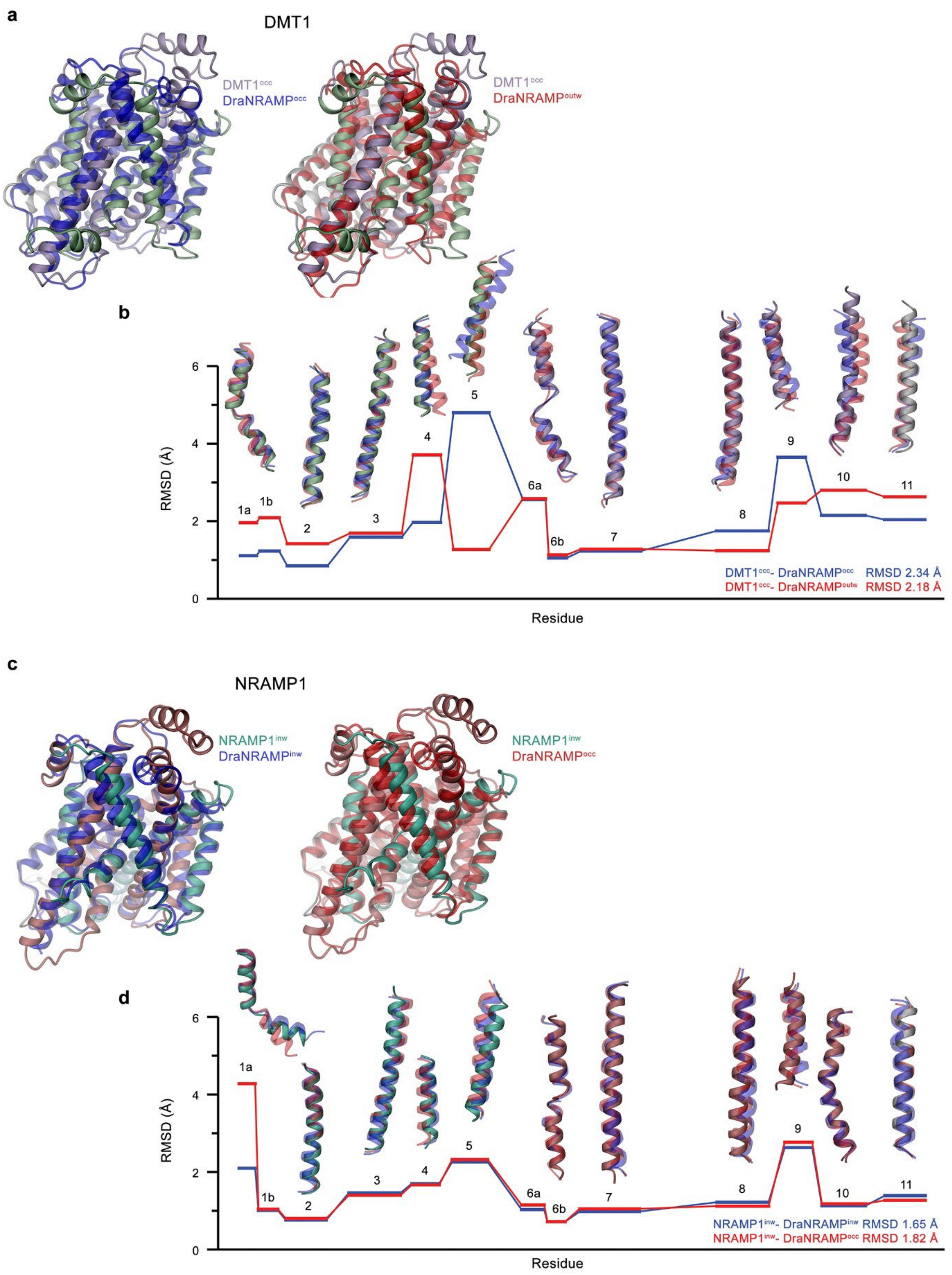

Supplementary Fig. 9: Analysis of SLC11 conformations. a Superposition of the occluded DMT1^{occ} structure with the inward-occluded DraNRAMP^{occ} (left, PDBID: 8E60) and the outward-facing DraNRAMP^{outw} structure of the point mutant G223W (right, PDBID 8E6N). **b** Averaged distances between Cα positions of indicated secondary structure elements in the superposition of DMT1^{occ} and DraNRAMP^{occ} (blue) and DraNRAMP^{outw} (red) obtained from the global superposition of the Cα

positions of the indicated 294 residues (yielding the listed overall RMSD value). Fit of the respective α-helices is shown above. Coloring is as in **a**. **c** Superposition of the inward-facing NRAMP1^{inw} structure with the inward-occluded DraNRAMP^{occ} (left, PDBID: 8E60) and the inward-facing DraNRAMP^{inw} (right, PDBID 8E6I). **d** Averaged distances between Cα positions of indicated secondary structure elements in the superposition of NRAMP1^{inw} and DraNRAMP^{occ} (red) and DraNRAMP^{inw} (blue) obtained from the global superposition of the Cα positions of the indicated 294 residues (yielding the listed overall RMSD value). Fit of the respective α-helices is shown above.

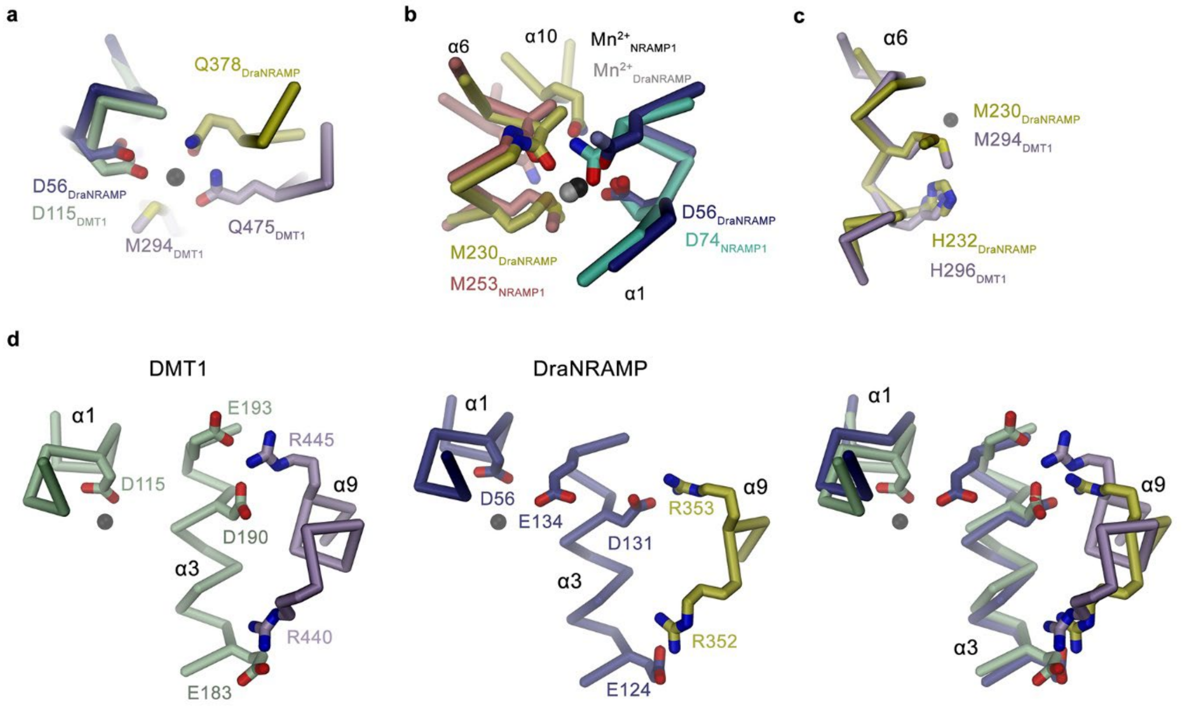

Supplementary Fig. 10: Features of the ion binding site. a Superposition of the ion binding sites in DMT1^{occ} and DraNRAMP^{occ}. The position of the ion bound to DMT1^{occ} is shown as black sphere. **b** Superposition of the ion binding sites in NRAMP1^{inw} and DraNRAMP^{inw}. The position of bound ions are shown as spheres (black NRAMP1^{inw}, grey DraNRAMP^{inw}). **c**, **d** Residues implied to play a role in H^+ transport. **c** Superposition of α6b with the Met that is part of the ion binding site and a conserved His located in intracellular direction displayed as sticks. **d** Ionic interaction network on α3 and α9 and its relationship to an Asp on α1 that is part of the metal ion binding site. Left, DMT1^{occ}, center DraNRAMP^{occ}, right superposition of both regions.

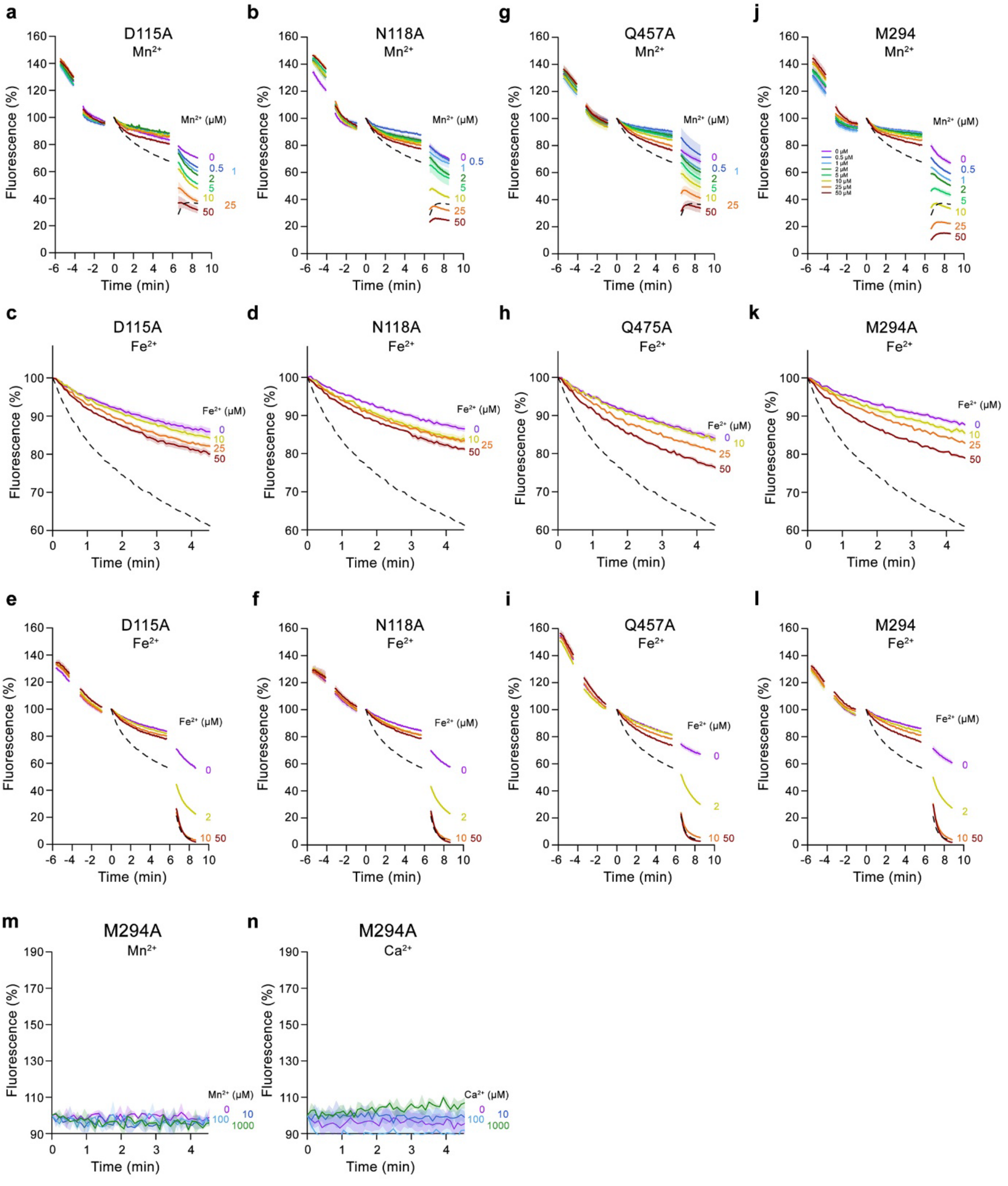

Supplementary Fig. 11: Functional properties of DMT1 mutants. a-f Mn^{2+} and Fe^{2+} transport by the DMT1 mutants D115A and N118A. **a**, **b** Full traces of transport experiments assaying the uptake of Mn^{2+} shown in Fig. 6b-c. **c**, **d** Section and **e**, **f** the corresponding full traces of transport experiments assaying the uptake of Fe^{2+}. **g-i** Mn^{2+} and Fe^{2+} transport by the DMT1 mutant Q457A. **g** Full traces of transport experiments assaying the uptake of Mn^{2+} shown in Fig. 6d. **h** Section and **i** the corresponding full traces of transport experiments assaying the uptake of Fe^{2+}. **j-l** Mn^{2+} and Fe^{2+} transport by the DMT1 mutant M294A. **g** Full traces of transport experiments assaying the uptake of Mn^{2+} shown in Fig. 6e. **h** Section and **i** the corresponding full traces of transport experiments assaying the uptake of Fe^{2+}. **a-l**

Time and concentration dependence uptake assayed by the quenching of the fluorophore calcein trapped inside the vesicles. **a, b, e**, **f, g, i, j, l** After two minutes of fluorescence recording an inwardly directed negative membrane potential was established by addition of the ionophore valinomycin (t=-4 min). Metal ions were added after equilibration of the signal (t=-30 s) and the transport mediated decay was recorded for six minutes (from t=0 to 6 min), before the addition of the ionophore calcimycin to equilibrate the metal ion concentration. **m**, **n** Addition of Mn^{2+} (**m**) or Ca^{2+} (**n**) to proteoliposomes containing the DMT1 mutant M294A did not show a pronounced influence on the fluorescence of Fura-2 trapped inside the vesicles, suggesting that there is no transport of either divalent metal ion. **a-l** Trace of WT upon addition of 50 µM Mn^{2+} is shown as dashed line for comparison. **a, b, g, j**, **n**, Data show mean of eight experiments from two independent reconstitutions. **c-f, h, i, k, l** Data show mean of four experiments from one reconstitution. **a-l, n** Errors are s.e.m. **m**, Data show mean of two experiments form one reconstitution for Mn^{2+} transport, Errors indicate range of data. **a-n** Fluorescence is normalized to the value after addition of substrate (t=0). Applied ion concentrations are indicated.

Supplementary Table 1: Kinetics

	DMT1 Fe^{2+}	DMT1 Mn^{2+}	NRAMP1 Fe^{2+}	NRAMP1 Mn^{2+}	EcoDMT Fe^{2+}	EcoDMT Mn^{2+}	DMT1 H^+	NRAMP1 H^+
K_m (µM)	2.5 ± 0.4	35.8 ± 8.7	1.4 ± 0.3	4.9 ± 1.0	8.3 ± 1.9	24.9 ± 2.9	6.1 ± 1.3	3.9 ± 1.5
v_{max} (dF min^{-1})	8.5 ± 0.4	11.4 ± 1.2	7.8 ± 0.4	7.7 ± 0.4	10.2 ± 0.8	16.1 ± 0.6	6.8 ± 0.3	2.5 ± 0.3
R^2	0.77	0.92	0.76	0.8	0.88	0.98	0.8	0.48

K_m and v_{max} are average of fits to the averaged measurements
Errors are s.d.

R^2 is calculated with values from individual measurements

Supplementary Table 2: Statistical Properties

	DMT1/Mn^{2+}	NRAMP1/Fe^{2+}	NRAMP1/Mn^{2+}	EcoDMT/Fe^{2+}	EcoDMT/Mn^{2+}
DMT1/Fe^{2+}	0.003 **	0.171 n.s.	0.1417 n.s.	0.1556 n.s.	<0.0001 ****
DMT1/Mn^{2+}	-	0.0025 **	0.0051 **	0.0064 **	0.0859 n.s.
NRAMP1/Fe^{2+}		-	0.0623 n.s.	0.1145 n.s.	<0.0001 ****
NRAMP1/Mn^{2+}			-	0.7412 n.s.	0.0002 ***
EcoDMT/Fe^{2+}				-	0.0248 *

$p>0.05$ n.s.; $p<=0.05$ *; $p<=0.01$ **; $p<=0.001$ ***; $p<=0.0001$ ****

Supplementary Table 3: List of primers.

Primer	Sequence (5'-3')
D115A forward	CGCCTATCTGGCCCCAGGCAACA
D115A reverse	ATAGACATCAGGAAGCCAGGG
N118A forward	GGACCCAGGCGCCATCGAGAGCG
N118A reverse	AGATAGGCGATAGACATCAG
M294A forward	AGCCGTGATCGCCCCACACAACATGTACCTG
M294A reverse	CCCACGATTCCCACTGCC
Q475A forward	GAATGTGCTGGCGAGCCTGCAGC
Q475A reverse	AGGAAATCGTTCATGCCTG

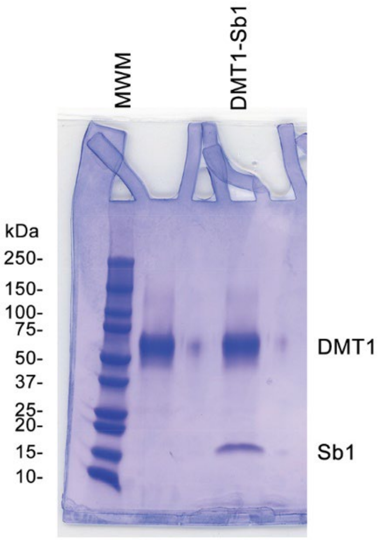

SDS PAGE gel for Supplementary Fig. 3b

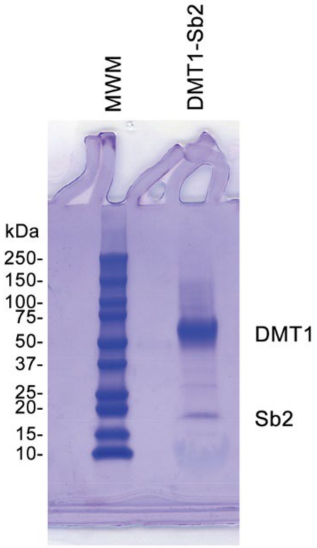

SDS PAGE gel for Supplementary Fig. 3c
